# Postpartum cardiac arrest in a woman with an uncorrected sinus venosus type of atrial septal defect: A case report

**DOI:** 10.1016/j.ijcchd.2023.100444

**Published:** 2023-02-09

**Authors:** Joelle Régine Mekoa Mbarga, Estelle Tenisch, Aicha Saleh, Mathieu Le Bloa, David Desseauve, Raymond Pfister, Giulia Domenichini, Christina Corby-Zauner, Jean-Francois Surmely, Judith Bouchardy, Etienne Pruvot, Tobias Rutz

**Affiliations:** a Service of Cardiology, Lausanne University Hospital and University of Lausanne, Lausanne, Switzerland; b Department of Radiology, Lausanne University Hospital and University of Lausanne, Lausanne, Switzerland; c Service of Obstetrics, Lausanne University Hospital and University of Lausanne, Lausanne, Switzerland; d Service of Cardiovascular Surgery, Lausanne University Hospital and University of Lausanne, Lausanne, Switzerland; e Service of Adult Intensive Care Medicine, Lausanne University Hospital and University of Lausanne, Lausanne, Switzerland; f Cabinet de Cardiologie Morges. Rue des Charpentiers 9, 1110, Morges, Switzerland

**Keywords:** pregnancy, Congenital heart disease, Cardiac arrest, Arrhythmias

## Abstract

The number of women with congenital heart disease (CHD) surviving to childbearing age is continuously growing. Although most pregnancies in this patient-population are well tolerated, there is a significant risk of potentially fatal complications. We describe a case of a previously completely asymptomatic patient who was diagnosed late during pregnancy with an inferior sinus venosus type atrial septal defect (ISV-ASD) and anomalous connection of all right pulmonary veins (PAPVC) who presented a cardiac arrest with ventricular fibrillation the day after delivery. She recovered completely and underwent subsequent surgical repair and implantation of a subcutaneous defibrillator (S-ICD).

A 28-year-old patient was referred at 33 weeks of gestation of her first pregnancy to a cardiologist to investigate a newly revealed systolic murmur. Her medical history and exercise capacity before and during pregnancy were unremarkable. There was no known cardiac condition in the family history. Blood oxygen saturation was >95% on room air and a systolic murmur on the second intercostal space at the left parasternal edge was present with a fixed split second heart sound. There were no signs of heart failure. The electrocardiogram (ECG) showed a complete right bundle branch block (Fig. 1A).

**Fig. 1:**
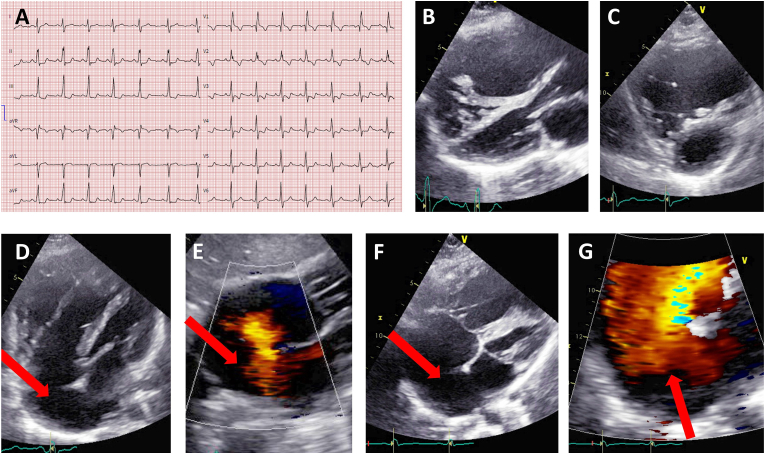
Resting ECG (A) and TTE at first presentation. (B) and (C): Parasternal long-axis and short-axis views showing the dilated RV. (D) 4-chamber (4CH), (E) subcostal, (F) and (G) modified parasternal short axis view (PSAX). Red arrows showing the ISV-ASD with/without color Doppler. (For interpretation of the references to color in this figure legend, the reader is referred to the Web version of this article.)

A transthoracic echocardiogram (TTE) revealed an inferior sinus venosus type atrial septal defect (ISV-ASD) with a left-to-right shunt, a severely dilated right ventricle (RV) and a diastolic D-shape of the interventricular septum (Fig. 1B–G, videos TTE 1–5). The RV systolic pressure was 30 mmHg. The tricuspid valve and the left ventricular systolic function were normal. The patient did not take any medication beside magnesium substitution for non-cardiac reasons.

Supplementary video related to this article can be found at https://doi.org/10.1016/j.ijcchd.2023.100444

The following are the supplementary data related to this article:

Multimedia component 1 1 Multimedia component 1

Multimedia component 2 2 Multimedia component 2

Multimedia component 3 3 Multimedia component 3

Multimedia component 4 4 Multimedia component 4

Multimedia component 5 5 Multimedia component 5

Due to the good tolerance of the pregnancy, a multidisciplinary board decided to closely follow the patient on an outpatient basis, plan a spontaneous vaginal delivery and propose a surgical repair at 6-month post-partum.

At 39 weeks and 6 days of gestation, our patient went into labor and delivered her baby after 6 hours through a vaginal delivery with no immediate complications. The amount of blood loss was 400ml and the Apgar score was 10.

On first day post-partum in the morning, while standing up for the first time, our patient described a sudden onset of unwellness followed by a transient loss of consciousness. After initially recovering our patient presented a cardiac arrest. There was no palpable pulse. Cardio-pulmonary resuscitation was immediately initiated. Ventricular fibrillation was diagnosed by an external defibrillator with sinus rhythm successfully restored after a single shock.

Blood tests showed no electrolytes disturbances. Whole-body computed tomography (CT) showed no pathological findings but the ISV-ASD and the anomalous return of all right-sided pulmonary veins (Fig. 2A–C, movies 3D_CT_1 and 2). Coronary angiography was unremarkable. Right-heart catheterization revealed normal pulmonary vascular resistance (PVR 0.81 WU). Arterial blood oxygen was 98%. Transesophageal echocardiography visualized the large ISV-ASD of 40 × 40mm. Cardiac magnetic resonance (CMR) calculated a Qp:Qs of 3.9–4.5 and confirmed the RV dilatation (RV enddiastolic volume, RVEDV 419ml) with a mildly reduced RV ejection fraction (RVEF 44%, Fig. 2D). There was no significant valvular disease. The tricuspid valve showed only a mild regurgitation. There was no myocardial scar. An electrophysiological study was not deemed necessary, as the further clinical course did not reveal any arrhythmias and the ECG remained unchanged. A genetic testing was not performed, however, could be considered to complete the work-up of potential causes of the patient's arrhythmic malignant event.

**Fig. 2:**
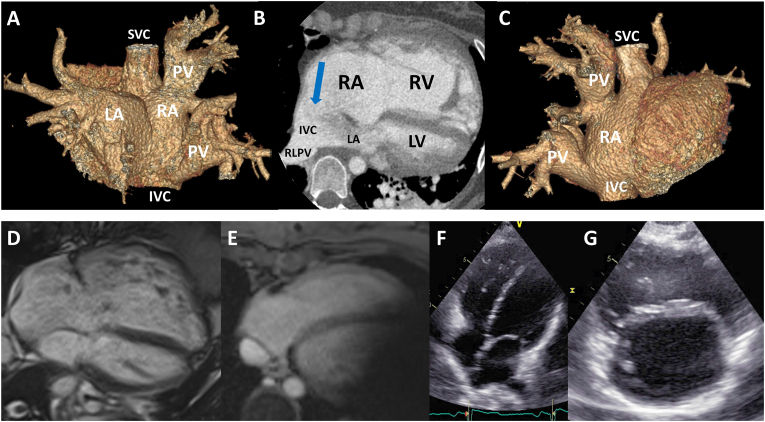
Posterior (A) and anterior (C) 3D reconstruction of CT of atria and anomalously connected right pulmonary veins draining into SVC, RA and IVC. (B) Modified 4CH view showing the ISV-ASD (blue arrow). (D) Cardiac magnetic resonance (CMR), 4CH showing the dilated RV. (E) CMR of RV post-repair with artifact of S-ICD. (F) and (G) Post-repair 4CH and PSAX showing the reduced RV size. Abbreviations: IVC: inferior vena cava, LA: left atrium, RV: right ventricle, LV: left ventricle, PV: pulmonary veins RA: right atrium, RLPV: right lower pulmonary vein, other see Fig. 1. . (For interpretation of the references to color in this figure legend, the reader is referred to the Web version of this article.)

Supplementary video related to this article can be found at https://doi.org/10.1016/j.ijcchd.2023.100444

The following are the supplementary data related to this article:

Multimedia component 6 6 Multimedia component 6

Multimedia component 7 7 Multimedia component 7

The patient subsequently underwent surgical repair and implantation of a subcutaneous internal cardioverter-defibrillator (S-ICD). At 6 months post-partum, the RV had recovered a normal size (RVEDV 127ml) with a mildly reduced RVEF (40%, Fig. 2E–G). Our patient reported an improved exercise capacity compared to the pre-pregnancy condition.

Diagnosis of an ASD during pregnancy is not rare. Although pregnancies in ASD patients are usually well tolerated, supraventricular arrhythmias are encountered as typical complications in addition to heart failure and paradoxical strokes [1]. In contrast, ventricular arrhythmias or cardiac arrest remain extremely uncommon [2,3]. The precise mechanism for the increased arrhythmia burden in pregnancies are not fully understood but is probably due to a combination of hemodynamic, hormonal, and autonomic changes [4]. The physiological adaption to match both maternal and fetal needs include a 40–50% increase in cardiac output due to increased heart rate and stroke volume. Elevated cardiomyocytes excitability, cardiac cavity enlargement and stretching of cardiomyocytes probably favor arrhythmias. Estradiol and progesterone tend to have pro-arrhythmic effects [4,5].

In addition to these physiological changes, in women with congenital heart disease (CHD) surgical scars and residual hemodynamic lesions augment further the already increased risk for arrhythmias.

The precise mechanism of the cardiac arrest of our patient remains speculative. The work-up did not reveal any myocardial scar, coronary artery dissection or pulmonary embolism. One hypothesis could be that the already severely dilated RV was unable to cope with the increased preload due to the uterine auto-transfusion after delivery. A further explanation could be that a vasovagal syncope when standing-up for the first time after delivery reduced the preload of the RV, resulting in a relative ischemia followed by ventricular fibrillation.

The question is whether we should have monitored our patient more closely, if a Holter monitor would have shown any arrhythmia or whether we should have even planned e.g., a cesarean section and correction of the ISV-ASD immediately after birth. According to the modified World Health Organization (mWHO) risk classification, an unrepaired ASD is associated with only a small increased risk for maternal mortality and our patient tolerated well the pregnancy not presenting any of the established risk factors for poor pregnancy outcome, all justifying our conservative strategy [1]. However, even if risk-stratifying scores are reassuring, potentially fatal complications can occur in pregnant CHD patients highlighting their need for a specialized follow-up during the pre-, peri- and postpartum period.

## Patient's consent

The patient has granted consent to publishing.

## Declaration of competing interest

The authors declare that they have no known competing financial interests or personal relationships that could have appeared to influence the work reported in this paper.
